# Cationic Liposomes as Vectors for Nucleic Acid and Hydrophobic Drug Therapeutics

**DOI:** 10.3390/pharmaceutics13091365

**Published:** 2021-08-30

**Authors:** Kai K. Ewert, Pablo Scodeller, Lorena Simón-Gracia, Victoria M. Steffes, Emily A. Wonder, Tambet Teesalu, Cyrus R. Safinya

**Affiliations:** 1 Materials, Physics, and Molecular, Cellular, and Developmental Biology Departments, and Biomolecular Science and Engineering Program, University of California at Santa Barbara, Santa Barbara, CA 93106, USA; victoria.ma.steffes@gmail.com (V.M.S.); eawonder@gmail.com (E.A.W.); 2 Laboratory of Precision- and Nanomedicine, Institute of Biomedicine and Translational Medicine, Centre of Excellence for Translational Medicine, University of Tartu, Ravila 14b, 50411 Tartu, Estonia; pablo.david.scodeller@ut.ee (P.S.); Lorena.Simon.Gracia@ut.ee (L.S.-G.); 3 Center for Nanomedicine and Department of Cell, Molecular and Developmental Biology, University of California at Santa Barbara, Santa Barbara, CA 93106, USA

**Keywords:** cationic liposomes, nucleic acids, nanoparticles, hydrophobic drug delivery, gene therapy, small-angle X-ray scattering, homing peptide, affinity targeting

## Abstract

Cationic liposomes (CLs) are effective carriers of a variety of therapeutics. Their applications as vectors of nucleic acids (NAs), from long DNA and mRNA to short interfering RNA (siRNA), have been pursued for decades to realize the promise of gene therapy, with approvals of the siRNA therapeutic patisiran and two mRNA vaccines against COVID-19 as recent milestones. The long-term goal of developing optimized CL-based NA carriers for a broad range of medical applications requires a comprehensive understanding of the structure of these vectors and their interactions with cell membranes and components that lead to the release and activity of the NAs within the cell. Structure–activity relationships of lipids for CL-based NA and drug delivery must take into account that these lipids act not individually but as components of an assembly of many molecules. This review summarizes our current understanding of how the choice of the constituting lipids governs the structure of their CL–NA self-assemblies, which constitute distinct liquid crystalline phases, and the relation of these structures to their efficacy for delivery. In addition, we review progress toward CL–NA nanoparticles for targeted NA delivery in vivo and close with an outlook on CL-based carriers of hydrophobic drugs, which may eventually lead to combination therapies with NAs and drugs for cancer and other diseases.

## 1. Introduction

**Figure 1:**
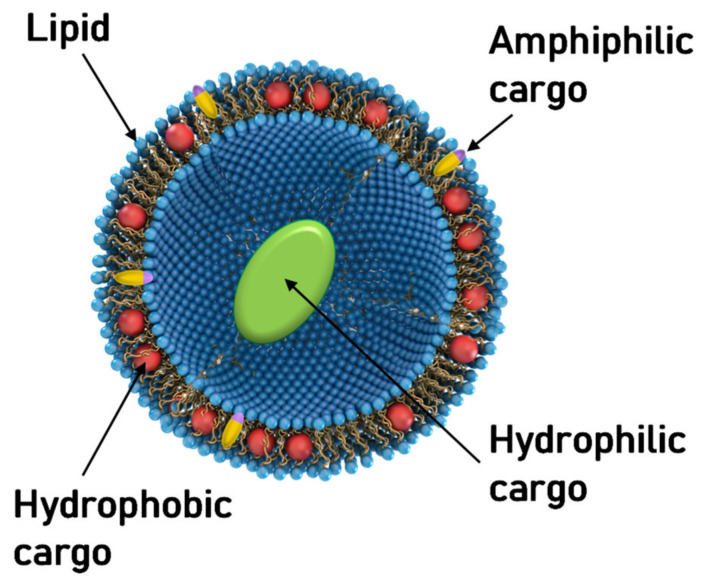
Schematic depiction of a unilamellar liposome consisting of a self-assembly of amphiphilic lipid molecules. The liposome can transport cargo at three distinct sites: within its hydrophobic bilayer (red spheres), within its hydrophilic aqueous interior (green ellipsoid), and at the amphipathic interface (yellow/blue rods).

Amphiphilic molecules, i.e., molecules with a polar, hydrophilic headgroup and a hydrophobic tail or tails, spontaneously self-assemble in water, primarily due to hydrophobic interactions [1]. The resulting assemblies expose the headgroups to the aqueous environment while avoiding exposure of the tails, which instead form their own hydrophobic environment. This understanding, which is now commonplace, originated only after Bangham and Horne discovered liposomes (also referred to as unilamellar or multilamellar vesicles; Figure 1) while studying suspensions of phospholipids by electron microscopy [2]. Liposomes, consisting of closed assemblies of bilayers of lipids, resembled cell membranes in the electron micrographs, thereby confirming that the makeup of these biological structures is lipid-based. Bangham and Horne’s work further demonstrated that lipid bilayer membranes can accommodate hydrophobic molecules while forming a barrier for polar, hydrophilic molecules. Thus, these studies also confirmed the hypothesis that the lipids of plasma membranes provide the permeability barrier that is essential to biological membrane function.

The structure of liposomes makes it evident that they can accommodate cargo at three different sites (Figure 1): their interior (hydrophilic), within the bilayer (hydrophobic), and at the interface (amphiphilic). Figure 1 schematically represents cargo at these sites as a green ellipsoid, red spheres, and yellow/blue rods, respectively. Soon, the potential of liposomes to deliver protein and small-molecule therapeutics was recognized and studied [3,4,5,6,7].

Only later, the use of cationic liposomes (CLs) to deliver nucleic acids (NAs) was pioneered by Phillip Felgner and coworkers [8,9] They demonstrated that positively charged CLs could simultaneously form complexes with negatively charged NAs and bind the anionic sulfate groups of cell-surface proteoglycans on mammalian cells [10], triggering not only uptake by cells but also the expression of the delivered DNA [8] and RNA [9]. Soon after the publication of the seminal papers by Felgner et al., the dominant structure proposed for CL–DNA complexes was a “bead-on-string” model of DNA strands decorated with distinctly attached CLs [11]. As described in detail below, CL–NA complexes instead spontaneously form a variety of novel self-assembled (liquid crystalline) structures [12,13,14,15] with distinct structure-activity relations [14,15,16,17,18].

In the wake of the landmark paper by Felgner et al., countless cationic lipids have been synthesized and studied [19,20,21,22,23,24,25,26,27,28,29,30,31]. In addition, numerous aspects of the formation and action of complexes of CLs with DNA and other nucleic acids (NAs) have been investigated [32,33,34,35,36,37,38,39,40,41,42,43]. These worldwide research efforts with CL vectors have the aim of improving efficacy both in vitro (at the cell level) and in vivo.

**Figure 2:**
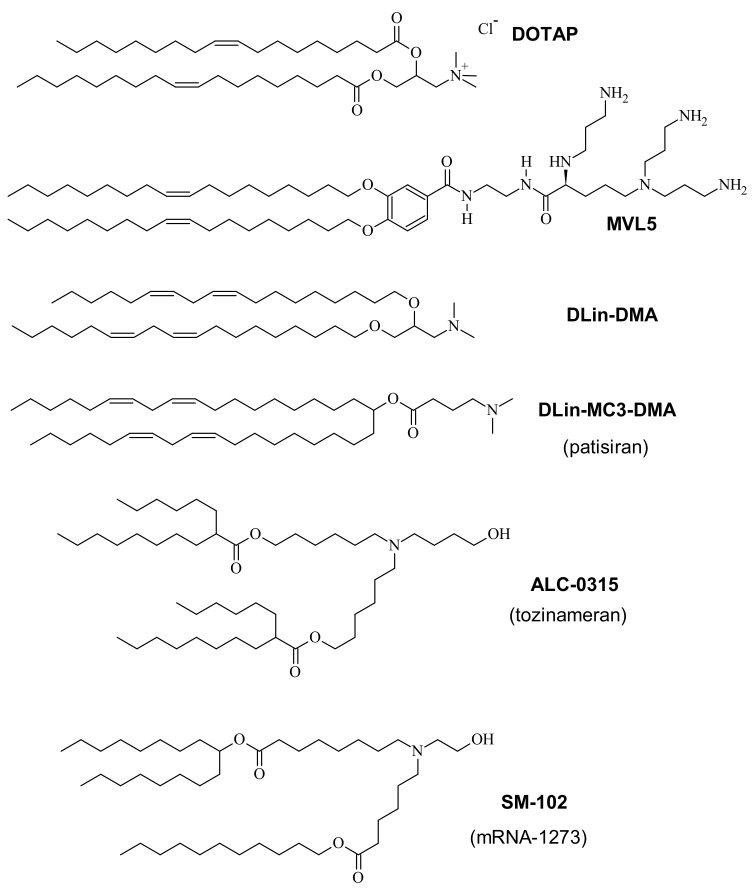
Structures of the cationic lipids DOTAP (1+; 1,2-dioleoyl-trimethylammonium propane chloride) [83], MVL5 (5+) [84], DLin-DMA [85], and the cationic lipids used in FDA-approved CL–NA nanoparticle formulations. Tozinameran and mRNA-1273 are names for the COVID-19 vaccines developed by Pfizer/BioNTech and Moderna, respectively. The positive charge on DOTAP is present independent of pH, while the amine groups of the other lipids acquire their charge by protonation below a certain pH. To highlight this fact, those lipids are sometimes called “ionizable” lipids. MVL5 is a commercially available multivalent ionizable lipid.

**Figure 3:**
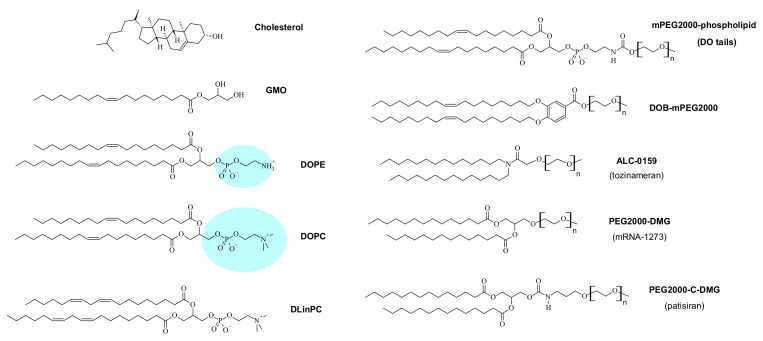
Structures of selected neutral (“helper”) lipids and PEG-lipids used in CL-based NA vectors. The molecular weight of the PEG chain of all depicted lipids is 2000 g/mol (*n* = 45). The phospholipids DOPC, DOPE, and DLinPC are zwitterionic, while GMO and cholesterol are uncharged. The blue highlighting represents the large difference in hydration for DOPE and DOPC. DLinPC: 1,2-dilinoleoyl-*sn*-glycero-phosphocholine; DO: dioleoyl; DOPC: 1,2-dioleoyl-*sn*-glycero-phosphocholine; DOPE: 1,2-dioleoyl-*sn*-glycero-phosphoethanolamine; GMO: glycerol monooleate.

CL-based vectors are now widely used for in vitro delivery of NAs, e.g., in functional genomics. However, the main motivation in the field has been the use of CL-based NA vectors in gene therapeutics [44,45,46], a concept explored in numerous clinical trials [45,47,48]. Major discoveries in the biochemical arena, such as gene silencing via RNA interference (RNAi) [49,50] induced by double-stranded small interfering RNA (siRNA) [18,51,52,53,54,55,56,57,58] and microRNA [59], gene editing via CRISPR/Cas-9 [60,61,62], and expression of base-modified mRNA [63,64,65,66,67,68,69,70], have greatly expanded the therapeutic potential of NAs. The first culmination of these efforts was the FDA approval of a lipid-based siRNA vector (patisiran/ONPATTRO^®^, Alnylam Pharmaceuticals) in 2018 for treatment of the polyneuropathy caused by hereditary transthyretin amyloidosis [71,72]. Patisiran delivers an siRNA targeting a conserved sequence within the transthyretin mRNA. Its action decreases hepatic production of mutant transthyretin to prevent the buildup of amyloid protein in peripheral nerves. Recently, the scale of this first breakthrough was dwarfed by the success and use (in potentially billions of patients) of two lipid-based mRNA vaccines [73] against SARS-CoV2. Building on decades of work on both the lipid vector [72,74,75] and the mRNA payload [70], the vaccines developed by Pfizer/BioNTech [76,77,78] and Moderna [79,80] became the first vaccines approved against COVID-19 in December 2020. The lipid compositions of the vaccine nanoparticles are similar to that of patisiran [81,82], albeit employing ionizable cationic lipids with branched rather than unsaturated tails and different PEG-lipids (Figure 2 and Figure 3).

With these successes, lipid-based synthetic NA vectors have stepped out of the shadow of viral vectors. Engineered viruses had been preferred in early clinical trials of gene therapy [47,48]. However, their use has resulted in severe side effects from immune reactions and insertional mutagenesis [86,87,88,89]. Furthermore, the finite capsid size of viral vectors limits the length of their NA cargo to a maximum of ≈20–30 kbp. Thus, engineered viruses are incapable of delivering larger human genes, let alone genes together with noncoding regulatory sequences (often exceeding 100 kbp) or human artificial chromosomes (>1 Mbp) [90,91,92]. In contrast, synthetic vectors such as CL–NA complexes are easier to produce, less immunogenic, and safer [93], and their formation by self-assembly imposes no fundamental limit on the size of the incorporated NA.

Without discounting the many breakthroughs that were required for their success, the recently approved CL–NA therapeutics represent low-hanging fruit in terms of delivery challenges. For example, there are no FDA-approved lipid-based vectors that incorporate elements for targeted delivery, which remains a major challenge. The development of efficient carriers of NAs remains the bottleneck for broadening their applications [53,94] and fully realizing the potential of gene therapy, and to do so will require major additional efforts in both basic and applied science. While a lot of progress has been made studying the mechanism of delivery and transfection by CL-based NA carriers [29,32,33,35,38,39,40,41,42,95,96,97], much more work remains before a comprehensive understanding of their interactions with membranes and other cell components such as the cytoskeleton can allow a rational design of effective vectors.

Establishing structure–activity relationships for cationic lipids for NA delivery is more challenging than for small molecule drugs. Instead of a single molecule interacting with, e.g., the binding pocket of a protein, a large number of lipids joins together in a dynamic assembly before interacting with the NA to form the active principle that engages with the target tissue or cell. In addition, the local environment may affect the CL–NA assembly and the interactions with its target. Thus, changes to the structure of the lipid may affect the efficacy of the NA vector in ways that are amplified by the process of self-assembly. With this review, we aim to provide a guide for parsing through the overwhelming number of lipids and formulations by relating the lipid structure to properties of lipid self-assembly. Some of these properties are only peripherally known to chemists designing these lipids because they stem from the biophysics and physical chemistry of lipids as well as from colloid science.

The importance of charge in preparing effective CL-based NA vectors was appreciated early on. In addition to the fact that complex formation with CLs protects NAs from degradation, an early rationale for utilizing cationic rather than neutral or negative liposomes to deliver DNA was that CL–DNA complexes designed to have an overall positive charge (i.e., with a lipid/DNA charge ratio > 1) would electrostatically adsorb to anionic mammalian cells, thus leading to cellular binding and more efficient uptake [8]. Here, the lipid/DNA charge ratio refers to the number of charges on the lipid divided by the number of charges on the DNA (under the conditions that the lipid and DNA are combined).

## 2. Lipid Shape and Membrane Curvature Elastic Energy Determine Their Self-Assembled Structures

**Figure 4:**
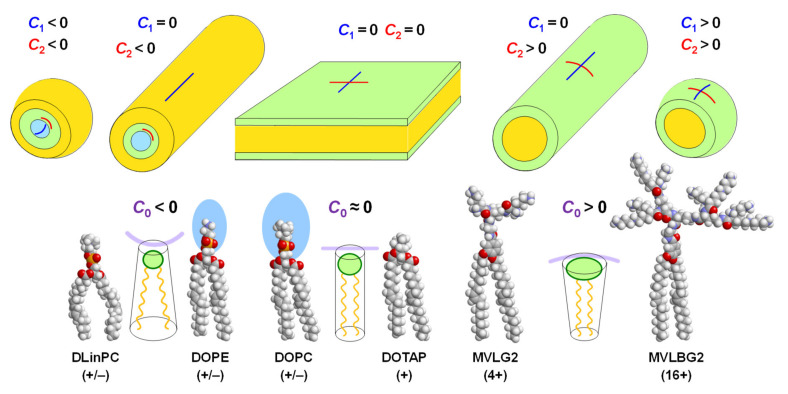
Relation of lipid shape and the resulting self-assemblies. Inverse-cone-shaped lipids give rise to inverse micellar structures (**left**); cylindrical lipids (**center**), where the headgroup area approximately matches the projected area of the tails, prefer (nearly) flat membranes (lamellar phases and large vesicles, depending on water excess); cone-shaped lipid (**right**) prefer micellar structures such as cylinders of varying length and spheres. Blue and red lines indicate the membrane curvatures *C*_1_ and *C*_2_, respectively, of the assemblies. Purple lines behind the schematic depictions of lipids with different shapes indicate the spontaneous curvature *C*_0_ of their assemblies. See Figure 2, Figure 3 and Figure 4 for the chemical structures of the displayed lipids.

It has been appreciated in surfactant and lipid science for over four decades that the shape of an amphiphile plays a major role in determining the structure of its self-assemblies [1,98,99,100,101]. Figure 4 illustrates the concept of lipid shape and the resulting assemblies with molecular models of a few lipids for illustration. The area of the lipid’s headgroup relative to that of the tails determines the lipid’s shape, and arrangement of these shapes with the constraint of exposing tails only to themselves and headgroups to the exterior aqueous environment naturally leads to the self-assembled structure. These assemblies, in turn, constitute the lipid building blocks of resulting CL–NA complexes.

A quantitative (but nonetheless semiempirical) parameter for describing lipid shape and the resulting assemblies is the “packing parameter”, *v*/*a*_0_*l*_c_. This dimensionless parameter is the ratio of the average area of the tails, expressed as the volume of the tails (*v*) over their maximum effective length (*l*_c_) and the optimal headgroup area (*a*_0_) [1]. For the lipids in Figure 4, the packing parameter increases from left to right, with *v*/*a*_0_*l*_c_ = 1 for lipids with a cylindrical shape. As an example of the usefulness of the packing parameter, consider the cationic lipids used in the COVID-19 vaccines (Figure 2) in comparison with hypothetical lipids of the same structure (i.e., the same *a*_0_) but without the branches in the tails. Adding branching will increase *v* while leaving *l*_c_ unaffected and thus decrease the packing parameter, meaning an increased propensity for the formation of the inverse structures depicted on the left in Figure 4.

The physical formalism to describe lipid membranes and their elastic properties uses the membrane curvatures, defined as the inverse of the membrane radii (*C*_i_ = 1/*R*_i_) in two orthogonal directions (Figure 4). For example, *C*_1_ = *C*_2_ = 0 for a flat membrane; *C*_1_ = *C*_2_ = 1/*R*_sph_ for a spherical membrane with radius *R*_sph_; and *C*_1_ = 0, *C*_2_ = *R*_cyl_ for a cylindrical membrane with radius *R*_cyl_. Negative curvature is also possible, e.g., for the surface of an inverse micelle (Figure 4, top left). The shape of a lipid determines the spontaneous (in other words, preferred) curvature of the membrane, termed *C*_0_. Introducing further the membrane modulus κ (a measure of the bending stiffness of the membrane) and the Gaussian modulus κ_G_ (a measure of the propensity for (κ_G_ > 0) or resistance to (κ_G_ < 0) forming saddle shapes, see Figure 6), the elastic free energy of a membrane per unit area, *E*/*A*, is

*E*/*A* = 0.5 κ (*C* − *C*_0_)^2^ + κ_G_ *C*_1_*C*_2_, (1)

with *C* = *C*_1_ + *C*_2_ being the mean curvature of the membrane and *C*_1_*C*_2_ being termed the Gaussian curvature of the membrane [102,103,104]. The elastic moduli κ and κ_G_ can be related to the interactions between neighboring lipids in the membrane using harmonic spring models [105,106].

The first term in Equation (1) describes the free energy cost of bending a membrane away from its spontaneous curvature, which increases as the membrane stiffness (κ) increases. As mentioned above, the shape of a lipid determines *C*_0_ [1,107]. Intuitively it may be easier to consider the spontaneous radius of curvature, *R*_0_, in some cases. Lipids with a headgroup area that is approximately equal to the projected area of their tails have a cylindrical shape. This corresponds to a spontaneous curvature *C*_0_ = 0 for the bilayer (Figure 4, center), and these lipids tend to assemble into lamellar bilayer structures such as lamellar lyotropic phases (at low water content) or large uni- and multilamellar vesicles (in excess water). Examples of such lipids are the neutral lipid DOPC (as well as other related phosphocholines with saturated tails) and the monovalent cationic lipid DOTAP. The molecular models in Figure 4 show that the steric size of the headgroup of DOTAP is much smaller than that of DOPC (or DOPE, see below), but the charge of the headgroup and the electrostatic repulsion between lipids that it induces as well as its hydration must be taken into account when considering the shape of the lipid in an aqueous environment.

The shape of a lipid with a headgroup area that is smaller than the tail area is described as an inverse cone. For lipids of this shape, *C*_0_ < 0, and they tend to form inverse micelles (Figure 4, left) that assemble further into structures such as the inverse hexagonal (H_II_) phase [107,108] for inverse cylindrical micelles or cubic phases for inverse spherical micelles. A well-known example of such lipids is DOPE, which differs from DOPC only in bearing an amino- instead of a trimethylammonium-group in its headgroup. This results in a larger hydration of the DOPC versus the DOPE headgroup and thus a difference in lipid shape (indicated by the blue color in Figure 3 and Figure 4), again highlighting that the structure of lipids must be considered in the context of their surroundings.

Instead of a decrease in headgroup size, an increase in the average cross-sectional area of the lipid tails can also change lipid shape from cylinder to inverse cone. An example of this is the lipid DLinPC, which bears two *cis* double bonds in its linoleoyl tails, compared to the single double bond in each tail of DOPC. As illustrated in Figure 4, the additional bend in the tail cannot be compensated for by gauche conformations in the rest of the chain, and the tail area increases. Consistent with this, CLs containing DLinPC showed a propensity to form the inverse hexagonal phase [109]. Similarly, increasing chain unsaturation facilitated the formation of the inverse hexagonal phase for a series of cationic lipids with C_18_ tails [85]. Of these lipids, the one with linoleyl tails (DLin-DMA; Figure 2), has a structure very similar to the cationic lipid used in the patisiran formulation (Figure 2, DLin-MC3-DMA; note the tails with two *cis* double bonds). It is intriguing to note in this context that the branching of the tails of the cationic lipids used in the mRNA vaccines against COVID-19 (Figure 2) also suggests that they have an increased cross-sectional area.

**Figure 5:**
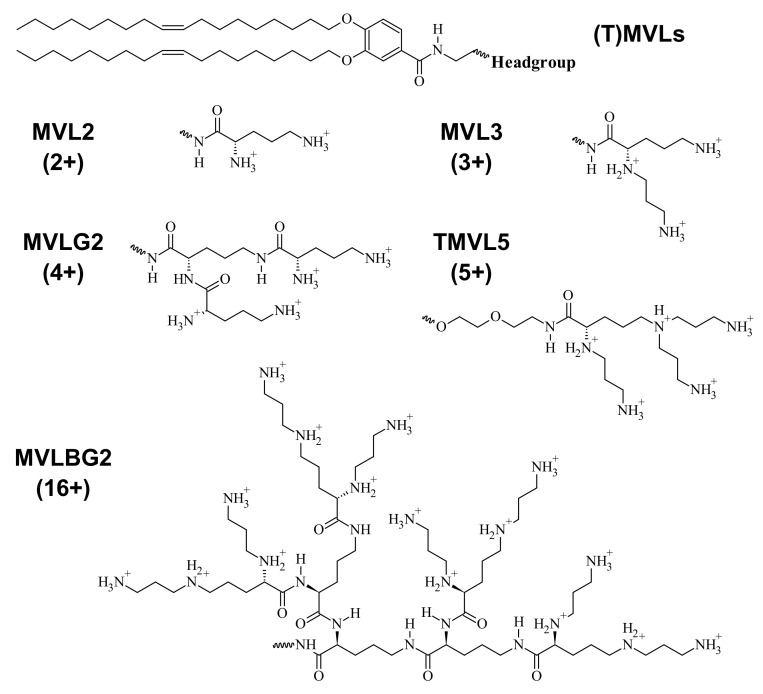
Chemical structure and maximum charge of custom synthesized multivalent lipids ((T)MVLs) mentioned in the text [14,17,30,84].

Cone-shaped lipids have a headgroup with an area that is larger than that of the tail. In this case, *C*_0_ > 0, and the lipids usually assemble into cylindrical or spheroidal micellar structures (Figure 4, right). This shape is common for amphiphiles with a single tail, but their high toxicity prevents their use in therapeutics. Lipids with two tails can be cone-shaped if they have a headgroup with a high charge and/or large steric size. An extreme example is the custom-synthesized lipid MVLBG2 [14,30] with a headgroup that bears 16 positive charges at full protonation (Figure 5). Lipids with a more moderate charge, such as DOGS [110] and MVL5 [111], have also shown a propensity for micellar structures. The headgroup of PEG-lipids is large because of the steric size of the polymer chain (depending on PEG length), and lipid mixtures containing them also form micellar structures, depending on the fraction of PEG-lipid (see below) [112,113,114].

As mentioned above, the environment of a lipid must be taken into account when considering its shape, and changing the conditions can lead to a change in shape and thus the structure of the lipid assembly. One example of this are changes in pH, which may increase or reduce the charge of a lipid’s headgroup, depending on the lipid structure. Another is the salt concentration in the aqueous environment; higher salt concentrations screen electrostatic interactions, effectively reducing the headgroup size.

The spontaneous curvature of a mixture of lipids is equal to the sum of the spontaneous curvatures of the individual lipids, *C*_0,*i*_, weighted with their molar fraction, *x*_i_, provided that complete mixing of lipids in the membrane takes place (see below). Thus, for a mixture of two lipids, *C*_0_ = *x*_1_*C*_0,1_ + *x*_2_*C*_0,2_, with *x*_i_ = *n*_i_/(*n*_1_ + *n*_2_) and *n*_1_, *n*_2_ the number of the two lipids in the membrane. An illustration of this is provided by mixtures of cone-shaped MVLBG2 with cylindrical DOPC. These mixtures form sheets (vesicles), cylindrical micelles that shorten with increasing content of MVLBG2, and spherical micelles at low, intermediate, and very high contents of MVLBG2, respectively [115].

An example on the other side of the curvature spectrum are mixtures of DOPE and DOPC, which form lamellar or inverse micellar structures depending on their composition [101].

**Figure 6:**
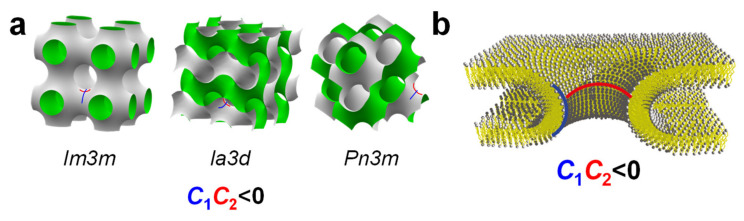
(**a**) Structures of a cubic phase with *Im3m* symmetry and two inverse bicontinuous cubic phases, the gyroid (Q_II_^G^, space group *Ia3d*) and the diamond (Q_II_^D^, space group *Pn3m*) cubic phases. These structures consist of a single continuous lipid bilayer interface (with saddle-shaped negative Gaussian curvature *C*_1_*C*_2_ < 0) dividing space into two disconnected water channels. The drawn surfaces, with one side colored gray and the other colored green, represent the shape of the water–bilayer interface [119,120]. (**b**) Schematic depiction of a membrane pore, illustrating its saddle-shaped negative Gaussian curvature. Adapted with permission from [121]. Copyright 2011 American Chemical Society.

The second term in Equation (1) contains the Gaussian modulus, κ_G_, and the Gaussian curvature, *C*_1_*C*_2_. Depending on the sign of their Gaussian modulus, membranes will prefer to form shapes of either positive or negative Gaussian curvature to minimize their elastic free energy. Examples of shapes with a positive Gaussian curvature (*C*_1_*C*_2_ > 0) are the outer (*C*_1_ > 0, *C*_2_ > 0) and inner (*C*_1_ < 0, *C*_2_ < 0) monolayer of a spherical vesicle, while flat bilayers (*C*_1_ = *C*_2_ = 0) and cylindrical micelles (*C*_1_ > 0, *C*_2_ = 0) have a Gaussian curvature of zero. Membranes with a positive Gaussian modulus κ_G_ > 0 will favor saddle-shaped surfaces with *C*_1_*C*_2_ < 0. These include the surfaces of bicontinuous cubic phases (Figure 6, left) and membrane pores (Figure 6, right; note the resemblance of the shape to a saddle) [103,107,116]. Thus, a consideration of membrane elasticity suggests that cubic phase-forming lipids (i.e., with κ_G_ > 0) favor the formation of pores (resulting from the fusion of two membranes that face each other in close proximity) [116,117,118]. Membrane fusion leading to pore formation has been mechanistically associated with the formation of bicontinuous cubic phases [116,117,118]. Because escape from the endosome after being internalized by the cell, which requires pore formation, is a major barrier to successful NA delivery, this is a highly relevant insight to the development of efficient CL-based NA vectors.

The Gaussian modulus (κ_G_) of the bilayer membrane may be driven positive in lipids with extreme inverse cone shapes. Simple elasticity theories, which treat the curvature elasticity of the flat lipid bilayer as the sum of the elasticities of two oppositely oriented monolayers, have shown that one may relate the Gaussian modulus of a bilayer to that of the monolayer via the equation [104,117]

κ_G_,_bilayer_ = 2κ_G,monolayer_ − 4δ*C*_0,monolayer_κ_monolayer_. (2)

Here, the bending rigidity of the bilayer κ_bilayer_ = 2κ_monolayer_, and δ is the monolayer thickness. The equation implies that lipids with inverse cone shape (C_0,monolayer_ < 0) contribute a positive term to the Gaussian modulus of the bilayer. Thus, one may drive a transition from the inverse hexagonal to the bicontinuous (inverse) cubic phase (κ_G_,_bilayer_ > 0) with lipids with sufficiently negative C_0_,_monolayer_. Support of this analysis is found in phase diagrams of lipid/water mixtures where the bicontinuous (inverse) cubic phase with κ_G_,_bilayer_ > 0 is often located near an H_II_ phase with C_0_,_monolayer_ < 0 [15,120,121]. Thus, designing vectors containing lipids with extreme inverse cone shapes (with a propensity for formation of bicontinuous cubic phases) may be considered a rational approach to overcoming the endosomal escape barriers of vectors because of the acquired fusogenic properties.

## 3. The Lamellar L_α_^C^ Phase of Cationic Liposome–DNA Complexes

While their opposite charge makes it appear intuitive that cationic liposomes and anionic NAs form complexes, the fact is that both already are associated with neutralizing counterions in solution. The primary driving force for their spontaneous self-assembly is the entropy gained by the release of positive counterions that are tightly bound to DNA (referred to as Manning condensation [122]) and negative counterions near the cationic liposome surface within the Guoy–Chapman layer [1]. As the cationic membranes neutralize the phosphate groups on the DNA, those small counterions gain in entropy because they are now free to diffuse and no longer bound to the DNA or cationic membranes. This driving force is also important in the assembly of other oppositely charged macro-ions, e.g., cationic polymers and DNA [123] or CLs and anionic proteins.

Initially, complexes of CLs and DNA were thought to consist of intact liposomes associated with DNA in a “bead-on-string” or “spaghetti and meatballs” structure [11]. Many subsequent studies have shown that such highly disordered assemblies are at best a short-lived intermediate in the process of CL–DNA complex formation. Ultimately, a complete topological transition from liposomes into collapsed condensates in the form of distinct liquid crystalline self-assemblies occurs [12,13,14,15,124]. The internal structure of the CL–DNA complexes as initially revealed by synchrotron small-angle X-ray scattering (SAXS) studies [12] is consistent with images from cryogenic electron microscopy studies [124,125,126].

**Figure 7:**
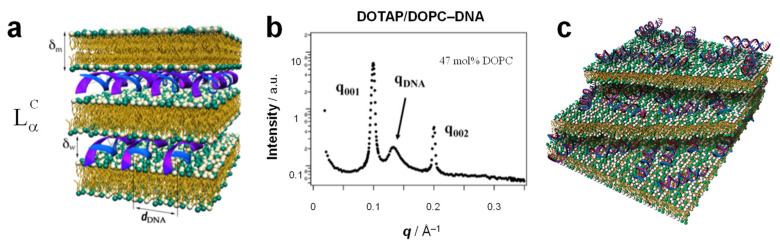
(**a**) Schematic of the L_α_^C^ phase of CL–DNA complexes, which consists of lipid bilayers of thickness δ_m_ alternating with DNA monolayers of thickness δ_w_. The interlayer spacing, which gives rise to the series of peaks labeled *q*_00i_ in SAXS, is *d* = δ_w_ + δ_m_ = 2π/*q*_001_. From [13]. Reprinted with permission from AAAS. (**b**) Example of the typical SAXS pattern resulting from CL–DNA complexes in the lamellar (L_α_^C^) phase. The Bragg reflections at *q*_001_ and *q*_002_ result from the multilamellar structure (see part **b**). The broad DNA–DNA correlation peak at *q*_DNA_ reflects the ordered arrangement of the DNA rods (see part **b**) with an average interaxial spacing *d*_DNA_ = 2π/*q*_DNA_. Complexes were formed from a DOTAP/DOPC (53:47, mol:mol) lipid mixture and λ-phage DNA. Reprinted with permission from [12]. (**c**) Schematic of the lamellar phase of CL–siRNA complexes. Note the lack of orientation order for the short siRNA rods. Reprinted with permission from [18]. Copyright 2007 American Chemical Society.

As mentioned above, membrane shape is often determined by the spontaneous curvature of the membrane, which is related to the molecular shape of the constituent lipids. The shape of the membrane component, in turn, often determines the structure of the resulting CL–NA complex. Bilayer sheets, reflecting a spontaneous curvature of *C*_0_=0, as building blocks give rise to the most common phase of CL–DNA complexes. This phase was also the first one to be discovered [12,124]: the lamellar, L_α_^C^, phase (Figure 7a), consisting of a multilamellar arrangement of DNA monolayers intercalated (sandwiched) between cationic membranes.

The characteristic signature of the lamellar phase in SAXS is a series of evenly spaced, sharp peaks at *q* = *q*_00L_, with *q*_00L_= L × *q*_001_. (Figure 7b) The lamellar spacing *d*, which comprises the thickness of the membrane (δ_m_) and the water gap (δ_w_), can be obtained from the position of the first peak as *d* = δ_m_ + δ_w_ = 2π/*q*_001_. Typical dimensions of a lamellar phase (measured for DOTAP/DOPC–DNA complexes [12]) are *d* = 64 Å, with δ_m_ = 39 Å [12] and δ_w_ = *d* − δ_m_ = 25 Å, which is approximately equal to the thickness of one monolayer of B-DNA (diameter ≈ 20 Å) including a hydration shell. (The membrane thickness was independently determined using SAXS of the lamellar L_α_ phase formed by the same lipids without DNA at an increasing volume fraction of water in the one-phase regime [127,128,129,130].)

In many cases, SAXS of the lamellar phase also reveals a broader peak at *q*_DNA_ (Figure 7b) that arises from DNA–DNA correlations and gives the spacing between the DNA rods as *d*_DNA_ = 2π/*q*_DNA_. Unlike the interlamellar spacing, the DNA spacing has been observed to vary widely depending on the composition of the membranes, in particular on the membrane charge density (due to the requirement of local charge neutrality in the complex [12]). The lower limit of *d*_DNA_ at high membrane charge density is ≈ 25 Å, corresponding to close packing of DNA chains with one hydration shell, but values as high as *d*_DNA_ ≈ 55 Å have been observed at low membrane charge density for DOTAP/DOPC–DNA complexes [12,131,132]. The DNA is electrostatically adsorbed on the cationic membrane, forming a two-dimensional (2D) smectic phase (i.e., a finite-size one-dimensional array of chains), as shown by quantitative line-shape analysis of the DNA correlation peak [133,134]. This makes the L_α_^C^ phase a hybrid liquid crystalline phase because the lipids form a 3D smectic phase while the DNA forms a 2D smectic phase.

The L_α_^C^ structure has been observed in CL–DNA complexes containing a broad variety of DNA: linear and circular (plasmid DNA) [16]. monodisperse (λ-phage DNA) [12] and polydisperse (e.g., calf thymus DNA) [135], long and very short [136]. Stable lamellar complexes form in water as well as cell culture medium [16] and disassemble only at very high salt concentrations (around 1 M monovalent salt, dependent on the membrane charge density) [131]. CLs formed from a wide variety of cationic lipids form the lamellar phase [21,137,138,139]. Even the strongly cone-shaped lipid MVLBG2 gives rise to L_α_^C^ complexes when mixed with a large amount of DOPC (90 mol%) [14].

NAs other than DNA also form lamellar phases. In the case of siRNA (≈19–27 bp RNA double strands with two nucleotide 3′-overhangs), no RNA–RNA correlation peak is observed, suggesting that the siRNAs are not ordered (aligned) within the aqueous layer (Figure 7c) [18]. When siRNA was replaced with similar short DNA confined between membranes of the L_α_^C^ phase, unexpectedly large end-to-end interactions between 11 bp DNA rods set in when their overhangs were reduced from 10 or 5 to 2 thymidines. This, in turn, led to the formation of a novel 2D columnar nematic liquid crystalline phase with finite-length columns consisting of stacks of on average four short DNA molecules [136].

Single-stranded mRNA, like DNA and short double-stranded siRNA, forms lamellar CL–NA complexes when complexed with DOPC/DOTAP membranes [140]. However, the interactions in CL–mRNA complexes are more complex compared with those in CL–DNA complexes in several aspects. Firstly, the electrostatic interactions of mRNA with cationic lipid membranes are biased by hydrophobic interaction of the exposed nucleic acid bases, and secondly, mRNA is more flexible and prone to form secondary structures than DNA [141], The exact structural features of mRNA in complexes with cationic lipid moieties remain undetermined. Nevertheless, the properties of lipids and CL–NA phases described in this review are expected to apply also to the understanding of the principles of self-assembly in CL–mRNA nanoparticles (NPs).

Other phases that are closely related to the L_α_^C^ phase but less relevant to delivery applications have also been reported. Three-dimensional columnar phases are characterized by the order of the DNA chains not only within a single layer but also between layers. Such phases have been observed for complexes of CL membranes in the “gel” phase with chain-ordered lipids with long DNA [142,143,144]. The additional ordering of DNA occurs due to charge-based DNA–DNA repulsion. Cationic lipids have reduced mobility in the gel phase, which limits their ability to screen electrostatic forces between DNA rods in different layers. Alternatively, the 3D ordering of DNA may be due to static membrane undulations that are coherent between layers. Recently, 3D long-range order was also observed for short, blunt DNA (which stacks into columns) confined between membranes containing lipids with chain-melted tails. This unexpected ordering is mediated by coherent membrane undulations across layers [145]. Theories of the lamellar phase of CL–DNA complexes also predict the existence of a novel “sliding columnar” phase, where the DNA rods have long-range orientation order from layer to layer but are not positionally correlated between layers [146,147,148].

## 4. The Inverse Hexagonal (H_II_^C^) Phase

**Figure 8:**
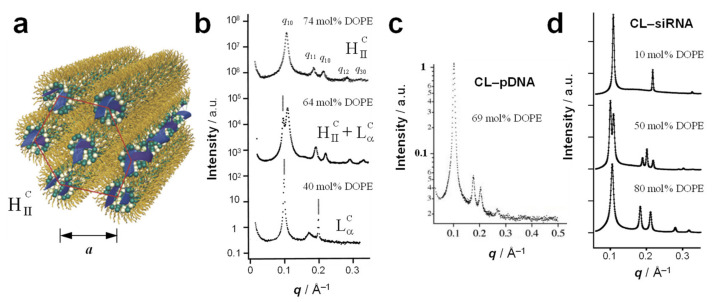
(**a**) Schematic of the inverted hexagonal (H_II_^C^) phase of CL–DNA complexes. In this phase, inverse cylindrical micelles containing DNA (i.e., DNA coated with a lipid monolayer) are arranged on a hexagonal lattice. The average spacing between the inverse micelles, *a*, can be obtained from the SAXS profile as *a* = 4π/√3*q*_10_. From [13]. Reprinted with permission from AAAS. (**b**) Example of the characteristic SAXS pattern of CL–DNA complexes in the H_II_^C^ phase (top profile). Also shown are characteristic SAXS patterns of CL–DNA complexes transitioning from the L_α_^C^ phase (bottom profile) to coexisting L_α_^C^ and H_II_^C^ phases (middle profile) and eventually the H_II_^C^ phase (top profile) as the content of DOPE in the membranes of the DOTAP/DOPE–DNA complexes increases. From [13]. Reprinted with permission from AAAS. (**c**) SAXS profile of complexes of DOTAP/DOPE (69 mol% DOPE) with plasmid DNA, revealing that the complexes are in the H_II_^C^ phase. Reprinted from [16], Copyright 2003, with permission from the Biophysical Society. (**d**) SAXS of DOTAP/DOPE–siRNA complexes reveals the formation of the lamellar phase at low content of DOPE (top), the inverse hexagonal phase at high content of DOPE (bottom), and coexistence of the two phases in a narrow regime of intermediate DOPE content. Reprinted with permission from [18]. Copyright 2007 American Chemical Society.

The inverse micellar building blocks resulting from lipids with preferred curvature *C*_0_ < 0, such as DOPE (Figure 4), favor the formation of the inverted hexagonal phase of CL–DNA complexes (Figure 8a). In this phase, termed H_II_^C^, DNA chains occupy the aqueous interior of inverse cylindrical micelles, and those micelles are arranged on a hexagonal lattice, forming a 2D columnar liquid crystalline phase.

The typical SAXS peaks arising from the 2D hexagonal lattice of H_II_^C^ CL–DNA complexes are labeled *q*_10_, *q*_11_, *q*_20_, *q*_12_, *q*_30_ (Figure 8b, 74 mol% DOPE). From these, the unit cell spacing may be calculated as *a* = 4π/√3*q*_10_. Given that evenly spaced peaks similar to those at *q*_10_, *q*_20_, *q*_30_, etc. are also observed for the lamellar phase, the existence of a peak at *q*_11_, i.e., *q*_10_ × √3 can be considered a key indicator for distinguishing the two phases. A typical unit cell spacing for the H_II_^C^ phase is *a* ≈ 67.4 Å (DOTAP/DOPE–DNA complexes with 74 mol% DOPE) [13]. Assuming a lipid monolayer thickness of ≈ 19.5 Å (half of δ_m_ above), this yields a diameter of the interior of the inverse micelles of ≈ 28 Å, sufficient for a DNA molecule with approximately two hydration shells.

The H_II_^C^ phase appears in the DNA complexes of DOTAP/DOPE CLs beyond a threshold content of DOPE (because for DOTAP alone, *C*_0_ = 0) [13]. As shown in Figure 8b, the SAXS profile exhibits only the features of the L_α_^C^ phase a DOPE content of 40 mol%, but at 74 mol% DOPE, a pure H_II_^C^ phase is observed (with the coexistence of the H_II_^C^ and L_α_^C^ phases at 64 mol% DOPE). These complexes were prepared from long, linear λ-phage DNA.

Complexes of DOTAP/DOPE CLs with reporter gene-carrying plasmid DNA (Figure 8c) [16] as well as with siRNA (Figure 8d) [18] also formed inverse hexagonal phases at sufficiently high membrane content of DOPE. This further supports the hypothesis that the membrane structure, guided by the lipid shape, is the main determinant of the structure of CL–NA assemblies. Similar to DOTAP/DOPE–DNA complexes (Figure 8b), varying the preferred curvature of the lipid mixture forming CL–siRNA by means of changing the ratio of DOTAP (*C*_0_ = 0) and DOPE (*C*_0_ < 0), makes it possible to steer the phase of the resulting CL–siRNA complexes toward lamellar (high percentage of DOTAP) or inverse hexagonal (high percentage of DOPE), with the coexistence of the two phases at an intermediate composition (Figure 8d) [18]. The SAXS profile of such a mixture of coexisting phases is a superimposition of the profiles of the phases, with the integrated intensity proportional to the amount of phase present in the sample.

## 5. Hexagonally Ordered Cylindrical Micelles Embedded in a DNA Honeycomb Lattice: The H_I_^C^ Phase

**Figure 9:**
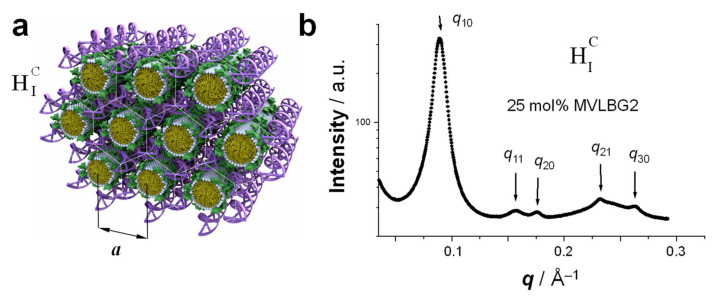
(**a**) Schematic of the H_I_^C^ phase of CL-DNA complexes. In this phase, cylindrical micelles formed by membranes containing strongly cone-shaped lipids such as MVLBG2 are arranged on a hexagonal lattice and surrounded by the oppositely charged DNA chains which form a honeycomb structure. (**b**) Synchrotron SAXS pattern of MVLBG2/DOPC–DNA complexes at 25 mol% of the highly charged lipid MVLBG2 (Figure 5). As described in the text, the SAXS peaks index to a 2D hexagonal lattice. Reprinted with permission from [14]. Copyright 2006 American Chemical Society.

When the lipid assemblies are cylindrical micelles (for lipids with positive preferred curvature, *C*_0_ > 0), CL–DNA complexes may form the hexagonal (H_I_^C^) phase (Figure 9a). In this liquid crystalline structure, the cylindrical lipid micelles form a 2D hexagonal lattice. They are surrounded by the DNA, which forms a three-dimensional continuous substructure with honeycomb symmetry [14]. This is an interesting contrast to the isolated DNA rods (1D) and sheets (2D) in the H_II_^C^ and L_α_^C^ phases, respectively (Figure 7a and Figure 8a).

The H_I_^C^ phase was first observed in a narrow composition range of CL–DNA complexes containing ≈25 mol% MVLBG2 (with the remainder DOPC) in their membranes. MVLBG2 is a highly charged (16+) multivalent cationic lipid with a dendritic headgroup [14,30]. A distorted H_I_^C^ phase was observed at higher contents of MVLBG2 and other highly charged (8+) dendritic lipids [115]. However, the headgroups of most other, even multivalent (up to 5+) cationic lipids, appear to be too small to force the formation of the cylindrical micelles that make up these phases.

The characteristic pattern of SAXS peaks of the H_I_^C^ phase (Figure 9b) resembles that of the H_II_^C^ phase (Figure 8b) because of their identical symmetry. However, the lattice spacing observed for the H_I_^C^ phase of MVLBG2/DOPC–DNA complexes is fairly large at *a* = 81.5 Å (compared to 67.4 Å for the H_II_^C^ phase of DOTAP/DOPE–DNA complexes, see above). The diameter of the hydrophobic core of the rod-shaped micelles is around 40 Å, as estimated from the thickness of a bilayer formed by lipids with identical (oleoyl) tails [14]. The DNA, headgroups, water, and counterions occupy the remaining space. For MVLBG2/DOPC–DNA complexes with higher contents (30–50 mol%) of MVLBG2, SAXS reveals a distorted hexagonal H_I_^C^ phase [115].

## 6. Cubic Lipid Phases with Embedded Nucleic Acid

**Figure 10:**
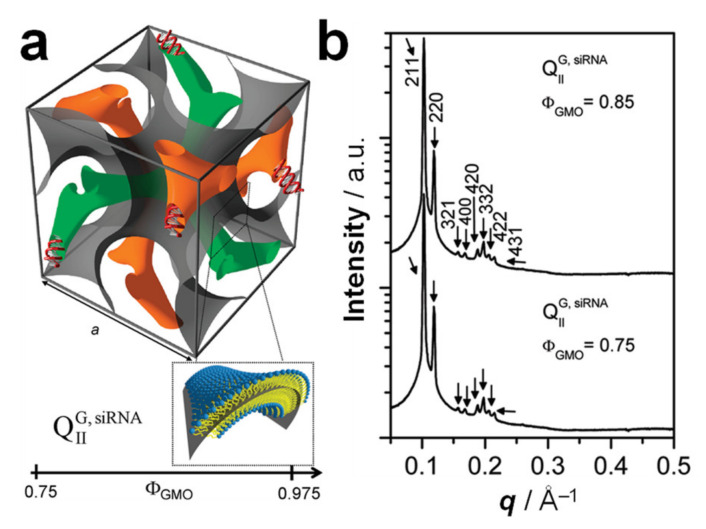
(**a**) Schematic depiction of the double-gyroid cubic phase of CL–siRNA complexes labeled (Q_II_^G, siRNA^). The two intertwined but independent water channels are shown in green and orange. For clarity, the lipid membrane separating the two water channels is represented by a gray surface corresponding to its center (see inset). Note the negative Gaussian curvature of the bilayer, *C*_1_*C*_2_ < 0. (**b**) Synchrotron SAXS data obtained for DOTAP/GMO–siRNA at a DOTAP/GMO molar ratio of 15/85 (top) and 25/75 (bottom). The large number of peaks reveals the body-centered gyroid cubic structure (space group *Ia3d*). Reprinted with permission from [15]. Copyright 2010 American Chemical Society.

In some cases, the incorporation of NA disrupts the preferred phase of the lipid component because it cannot otherwise be accommodated in the assembly. For example, the lipid GMO [149] (Figure 3) readily forms bicontinuous cubic phases such as the gyroid Q_II_^G^ with space group *Ia3d* (Figure 6a); this holds true even when GMO is mixed with (relatively small amounts of) cationic lipids such as DOTAP or MVL5 [15,121,150]. Both of these cubic phases with added cationic lipid are able to incorporate functional siRNA to generate a novel bicontinuous double gyroid cubic lipid phase, with the siRNA residing its two water channels (Figure 10a) [15,121]. The name of the phase indicates the fact that both the water and the lipid subphases are continuous in three dimensions, and that there are two independent water channels of gyroid symmetry (*Ia3d* space group). Figure 10b shows SAXS profiles for two compositions, illustrating the large number of peaks observed for this phase. For this phase, the lattice spacing *a*_Q_ = 2π√6/*q*_211_. A gyroid cubic structure has also been proposed for siRNA complexes of a mixture of GMO with divalent cationic gemini lipids [151]. More recently, cubic CL–siRNA with *Im3m* symmetry have been discovered when a small amount of PEG-GMO lipid was added to DOTAP/GMO mixtures at very high GMO content (95 mol%) [152,153].

When CLs of the compositions that form double-gyroid cubic CL–siRNA complexes are mixed with DNA, complexes in the H_II_^C^ phase are formed. This likely occurs because the energetic cost of bending the DNA to conform to the highly curved channels of the double-gyroid phase is greater than the cost of rearranging the lipid phase into the related H_II_^C^ phase. In fact, studies with a series of short DNA duplexes of varied length and end structure (“sticky”, repulsive, and no overhangs) revealed that stacking of these short pieces of DNA can induce the H_II_^C^ phase. Thus, counteracting the stacking with repulsive (nonpairing) overhangs or increased temperature shifted the equilibrium towards the gyroid cubic phase [154].

## 7. Transfection Efficiency and the Structure of CL–DNA Complexes

### 7.1. The Early Rise of DOPE and Its Relation to Complex Structure

**Figure 11:**
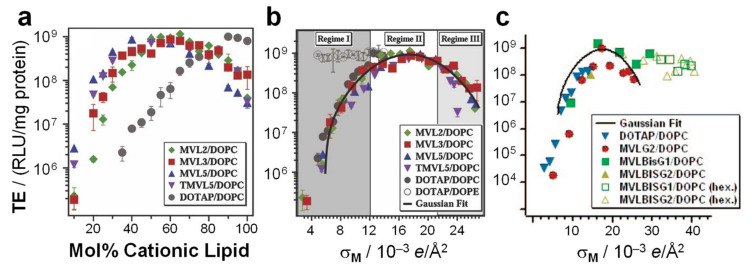
The membrane charge density (σ_M_, the average charge per unit area of the membrane) is a universal parameter for the transfection efficiency (TE) of lamellar CL–DNA complexes, but not for nonlamellar (H_II_^C^ or H_I_^C^) complexes. (**a**) TE plotted as a function of molar fraction cationic lipid for DNA complexes of MVL2, MVL3, MVL5, TMVL5, and DOTAP mixed with DOPC (see Figure 2, Figure 3 and Figure 5). (**b**) The same data as in (**a**), but plotted as a function of σ_M_ collapses onto a universal, bell-shaped curve as a function of σ_M_ (the solid line is a Gaussian fit to the data). TE data for DOTAP/DOPE complexes (open circles, H_II_^C^ phase) deviates from the universal curve, indicative of a distinctly different transfection mechanism for the inverted hexagonal phase. Three regimes of transfection efficiency are highlighted as described in the text. The membrane charge density can be written as σ_M_ = [1 − Φ_nl_/(Φ_nl_ + *r*Φ_cl_)]σ_cl_. Here, *r* = *A*_cl_/*A*_nl_ is the ratio of the headgroup areas of the cationic and the neutral lipid; σ_cl_ = *eZ*/*A*_cl_ is the charge density of the cationic lipid with valence *Z*; Φ_nl_ and Φ_cl_ are the mole fractions of the neutral and cationic lipids, respectively. The membrane charge density was calculated using *A*_nl_ = 72 Å^2^, *r*_DOTAP_ = 1, *r*_MVL2_ = 1.05 ± 0.05, *r*_MVL3_ = 1.30 ± 0.05, *r*_MVL5_ = 2.3 ± 0.1, *r*_TMVL5_ = 2.5 ± 0.1, *Z*_DOTAP_ = 1, *Z*_MVL2_ = 2.0 ± 0.1, *Z*_MVL3_ = 2.5 ± 0.1, *Z*_MVL5_ = *Z*_TMVL5_ = 4.5 ± 0.1 [17]. (**c**) TE for DNA complexes of MVLG2 (4+), MVLBisG1 (8+), MVLBisG2 (16+), and DOTAP mixed with DOPC (see Figure 2, Figure 3 and Figure 5) plotted as a function of membrane charge density. Filled symbols are for lamellar complexes, while empty symbols are for complexes in the H_I_^C^ or distorted H_I_^C^ phases. Again, the data for nonlamellar complexes deviates from the universal curve for lamellar complexes, indicating different transfection mechanisms. Parts (**a**,**b**) adapted from [17] with permission from John Wiley & Sons, Ltd. Part (**c**) adapted with permission from [115]. Copyright 2009 American Chemical Society.

Early studies revealed that CL–DNA complexes displayed increased transfection efficiency (TE) if the cationic lipid was combined with a neutral lipid. (TE is a measure of the extent of expression of an exogenous gene that is transferred into the cell). The term “helper lipid” was coined for these lipids, and DOPE, probably initially chosen because of its reputation as a “fusogenic” lipid, proved much more successful than DOPC [155]. Considering the propensity of DOPE for inverse hexagonal structures, this suggested a correlation between the liquid crystalline structure of CL–DNA complexes and their TE. A series of studies designed to elucidate this correlation indeed found important structure-dependent differences. As a function of increasing the content of cationic lipid, the TE of the DNA complexes of a series of cationic lipids mixed with DOPC increased to a maximum level before decreasing again (Figure 11a). At its maximum, the TE of these lamellar complexes was the same as that of DOTAP/DOPE complexes in the H_II_^C^ phase. However, in terms of the ratio of cationic lipid to helper lipid, the position of the maximum in TE shifts (Figure 11a), depending on the charge of the cationic lipid and the size of its headgroup. This means that it is easy to miss the most effective composition when only testing a few ratios of cationic to helper lipids, as was done in early investigations. In contrast, the TE of CL–DNA complexes in the H_II_^C^ phase is large over a wide range of compositions (Figure 11b). This means that the early investigations were very likely to find a cationic lipid/DOPE mixture with high TE, even if only a few compositions were tested.

### 7.2. Membrane Charge Density as a Universal Parameter for Transfection by Lamellar CL–DNA Complexes

Importantly, the work described above also revealed that the membrane charge density, σ_M_, is a universal parameter predictive of the TE of lamellar CL–DNA complexes [16,17]. The membrane charge density is defined as the average charge per unit area of the membrane. It can be considered a lipid-independent way to describe how highly charged a membrane is. In contrast, variations in headgroup charge and size mean that membranes at the same mol fraction of cationic lipid can carry very different numbers of charges per area. Figure 11a,b illustrates how the membrane charge density unifies the dependence of TE on composition. Figure 11a plots the TE against the molar fraction of cationic lipid, for DNA complexes of monovalent DOTAP and a series of custom-synthesized multivalent lipids ((T)MVLs, Figure 5) with systematically varied headgroup charge (between 2+ and 5+) mixed with DOPC [17,84] The amount of DNA and the charge of cationic lipid to DNA were kept constant for all data points (at an optimal charge ratio of 2.8). While the shape of the curves is similar, the maxima appear at different molar ratios of cationic lipid/DOPC. In contrast, when the TE of the complexes is plotted against the membrane charge density σ_M_ (Figure 11b), the data merge onto a single (universal) curve. This identifies σ_M_ as a universal parameter for transfection by lamellar CL–DNA complexes (i.e., a predictor of TE). The solid curve in Figure 11b is the fit of a Gaussian curve to the TE data which yields the optimal charge density σ_M_* = 17.0 ± 0.1 × 10^–3^ e/Å^2^ (see the caption of Figure 11 for the fitting equation) [17].

The shape of the universal curve suggests three distinct regimes of TE for lamellar CL–DNA complexes (highlighted by different backgrounds in Figure 11b), which can be related to two barriers to transfection in a simple model to explain the data. At low membrane charge density, an increase in σ_M_ (regime I, dark gray) enhances fusion between the cationic membranes of lamellar complexes (trapped in endosomes) and anionic endosomal membranes. This facilitates the release of complexes into the cell cytoplasm and thereby transfection. In contrast, at very high membrane charge density (regime III, light gray), an increase in σ_M_ progressively inhibits cationic membranes from efficiently releasing the DNA following an endosomal escape. These opposing trends give rise to the optimal regime II around σ_M_*, where σ_M_ is large enough to promote fusion with and escape from the endosome but not too large to prevent the subsequent release of DNA from the complex [17].

In contrast to the strong dependence of the TE of lamellar CL–DNA complexes on σ_M_, nonlamellar complexes show high TE independent of σ_M_. This is true both for H_II_^C^ complexes (Figure 11b, open circles, DOTAP/DOPE–DNA complexes) [16,17] and complexes in the H_I_^C^ and distorted H_I_^C^ phases [14,115] (Figure 11c) and indicates that their mechanism of action must be distinct from that of lamellar complexes, including different interactions of the complexes with cellular membranes and organelles [16,17]. For example, the high TE of H_II_^C^ complexes in regime I is likely related to the efficient fusion between the membranes of complexes containing DOPE and cellular membranes (e.g., those of the endosome) [16].

Note that because of the high content of DOPE required to induce the phase, only the regime of low σ_M_ is accessible with H_II_^C^ complexes. In contrast, the high charge of the lipids that form the H_I_^C^ and distorted H_I_^C^ phases mean that these phases only appear at high σ_M_. (At low contents of the same lipids, *C*_0_ is dominated by that of DOPC and lamellar phases are formed.) The contiguous substructure of DNA in these assemblies may be the reason that their efficiency remains high even at extremely high membrane charge density.

### 7.3. Highly Efficient Gene Silencing with Cubic CL–siRNA Complexes

As mentioned above, cubic phase forming lipids favor saddle-splay-shaped surfaces with negative Gaussian curvature (*C*_1_*C*_2_ < 0) which are also found in membrane pores. Pore formation is a possible mechanism for escaping the endosome after endocytosis of complexes, a rate-limiting step in transfection [156,157,158]. In fact, for a series of cationic phospholipids with varied tails, the difference in their propensity to form cubic phases with lipids found in cellular membranes has been invoked as a possible explanation of the differences in transfection efficiency [21,138,139]. Another approach was based on the hypothesis that cubic-phase CL–NA complexes would exhibit enhanced membrane fusion with cellular membrane barriers such as endosomal membranes because the constituting lipids promote the formation of pores with the desired negative Gaussian curvature [15,116,121]. The enhanced fusion, in turn, would result in efficient cytoplasmic siRNA delivery and thus gene silencing.

**Figure 12:**
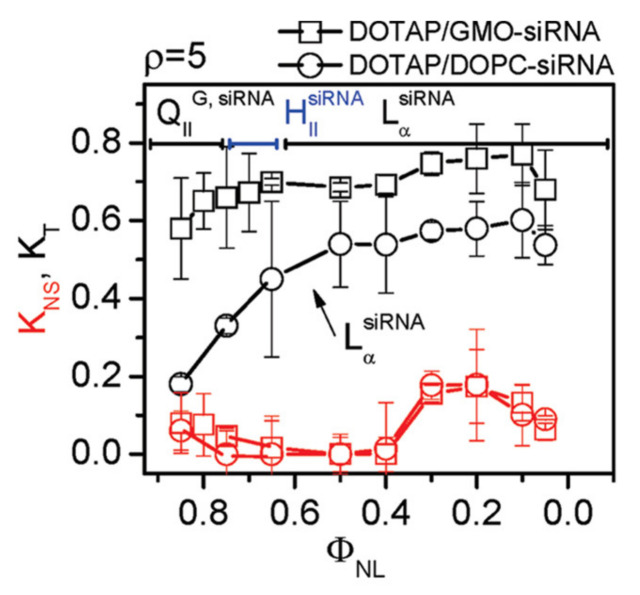
Sequence-specific gene silencing of CL–siRNA complexes incorporating the cubic-phase forming lipid GMO (Figure 2) is strongly improved compared to DOTAP/DOPC–siRNA complexes. Optimal silencing corresponds to K_T_ (total (specific and nonspecific) gene knockdown; black lines and symbols) approaching 1 while K_NS_ (nonspecific gene knockdown, red lines, and symbols) is minimal. DOTAP/GMO–siRNA complexes (squares) are in the gyroid cubic phase (Q_II_^G,siRNA^) at a high mole fraction of neutral lipid (Φ_NL_) where K_T_ is high and K_NS_ is low. In contrast, lamellar (L_α_^siRNA^) DOTAP/DOPC–siRNA complexes (circles) show low K_T_ at high Φ_NL_. The increased K_NS_ at low Φ_NL_, when both formulations form lamellar complexes because of their high content of DOTAP (*C*_0_ ≈ 0), indicates an undesirable onset of vector toxicity. Reprinted with permission from [15]. Copyright 2010 American Chemical Society.

The cubic gyroid phase of CL–siRNA complexes indeed turned out to be capable of efficiently delivering siRNA, resulting in sequence-specific gene silencing without toxicity even at very low membrane charge density (Figure 12) [15,121]. This is in contrast to lamellar CL–siRNA complexes, which require relatively high membrane densities to enable endosomal escape, and supports the idea that a different mechanism of fusion is operating. The low cytotoxicity is notable because DOPE, which forms the nonlamellar H_II_^siRNA^ phase at appropriate membrane compositions, exhibits significant toxicity at the high amounts of lipid required for siRNA delivery [18].

## 8. From In Vitro to In Vivo

An early major challenge to successful drug delivery with liposomes in vivo was their rapid clearance from circulation by a component of the immune system called the mononuclear phagocytic system [159,160,161]. A solution to this challenge are so-called STEALTH^®^ liposomes, whose surface is covered by poly(ethylene glycol) (PEG), a hydrophilic polymer, typically at a molecular weight (MW) of 2000 or 5000 [162,163,164,165,166,167,168,169,170]. To anchor it to the surface of the liposome, the PEG is attached to a lipid moiety, creating a PEG-lipid (Figure 3). These lipids were evolved from glycosphingolipids with sialic acid groups [171,172], used in the earliest attempts to develop long-circulating liposomes by mimicking red blood cells.

The PEG-lipid sterically stabilizes STEALTH liposomes, meaning that the shell of hydrophilic polymer acts as a physical barrier to objects approaching the liposomes. The utility of this repulsive interaction (with a range on the scale of the size of the polymer chain [173,174,175]) is broad: it counteracts both electrostatic adhesion of oppositely charged particles to cationic or anionic liposomes [176] and the nonspecific adhesion of, e.g., proteins or other liposomes due to van der Waals interactions [160,162,163]. Accordingly, PEGylated liposomes exhibit extended in vivo circulation times (suggesting that PEGylation limits adhesion of blood plasma opsonins, a necessary event for removal by immune cells), and multiple formulations are now FDA-approved medicines. A prominent example is Doxil^®^, a liposomal formulation of doxorubicin, for cancer chemotherapy [177].

As mentioned earlier, complexation with NAs completely disrupts and rearranges the structure of CLs, independent of whether bilayer- (e.g., L_α_^C^) or nonbilayer-type (e.g., H_II_C or H_I_^C^) CL–NA complexes form. These complexes are prone to aggregation in salt solution (e.g., cell culture medium). However, it is possible to use PEG-lipids to sterically stabilize CL–NA assemblies to sizes around 50–200 nm [55,126,178,179,180,181,182,183]. One way of achieving this is to use PEGylated CLs for complexation with NAs in an aqueous environment, where the specifics of the preparation method control important characteristics of the resulting CL–NA nanoparticles (NPs) [126,178,184]. Another preparation method combines an ethanolic solution of lipids with an aqueous solution of NA that is at a pH at which the ionizable CLs used are protonated, preferably using a microfluidic mixer. This method yields CL–NA NPs that have been termed LNPs after a dialysis step to remove residual ethanol and adjust the pH of the LNP suspension to physiological pH [55,181,182,183]. This is the method used for the preparation of patisiran and the CL-based COVID-19 vaccines.

The internal structure of CL–NA NPs again depends on the lipid composition: the formation of lamellar structures has been observed with SAXS and cryo-EM [126,178], as has the absence of periodic ordering (which makes structure determination with SAXS difficult) [126], in particular for LNP formulations that contain cationic lipids with a propensity for forming nonbilayer H_II_ phases [185] and large amounts of cholesterol [183,186].

To avoid opsonization and nonspecific attachment to cells (essential for targeted in vivo applications) the surface of CL–NA NPs requires a PEG-lipid concentration that is high enough to ensure that the PEG chains are in the brush regime (i.e., stretched-out chains; e.g., 10 mol% PEG2000-lipid) [170,173,174,175,187]. Unfortunately, this extent of PEGylation strongly reduces the transfection efficiency of the CL–DNA NPs by increasing the barriers for both cell attachment and endosomal escape [179,180,184]. It is therefore important to devise strategies that can counter these undesirable effects of PEGylation.

**Figure 13:**
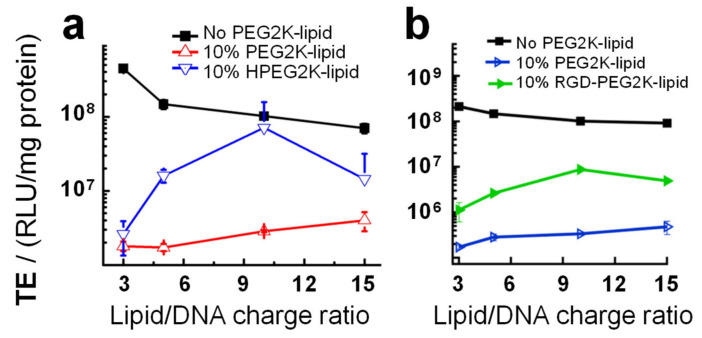
Two strategies to enhance transfection efficiency (TE) of PEGylated CL–DNA complexes. TE in murine CCL-1 cells is plotted versus *ρ* (lipid/DNA charge ratio) for DOTAP/DOPC/PEG-lipid–DNA complexes (80 mol% DOTAP(1+)) and control complexes without PEG-lipid. TE drops strongly upon the inclusion of 10 mol% PEG-lipid. However, complexes containing RGD-PEG2K-lipid or HPEG2K-lipid instead show partial recovery of TE, which is due to distinct mechanisms as discussed in the text. (**a**) Comparison of the TE of complexes without PEG2K-lipid (black), with 10% PEG2K-lipid (red), and with 10 mol% acid-labile HPEG2K-lipid (blue). Adapted from [179], Copyright 2012, with permission from Elsevier. (**b**) Comparison of the TE of complexes without PEG2K-lipid (black), with 10% PEG2K-lipid (blue), and with 10 mol% RGD-PEG2K-lipid (green). Adapted from [180], Copyright 2014, with permission from Elsevier.

**Figure 14:**
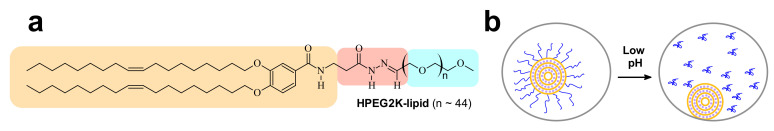
(**a**) Structure of the HPEG2K-lipid. The acid-labile acylhydrazone moiety is underlain in red, the lipophilic tails in tan, and PEG in blue. (**b**) Schematic depiction of the proposed mechanism of TE recovery by the low-pH-sensitive HPEG2K-lipid. During the maturation of endosomes, acidification cleaves the PEG chains from the lipid tails. This unmasks the positive charge of the CL–DNA NP, allowing electrostatically mediated recruitment to, and fusion with, the negatively charged endosomal membrane, facilitating endosomal escape [179].

**Figure 15:**
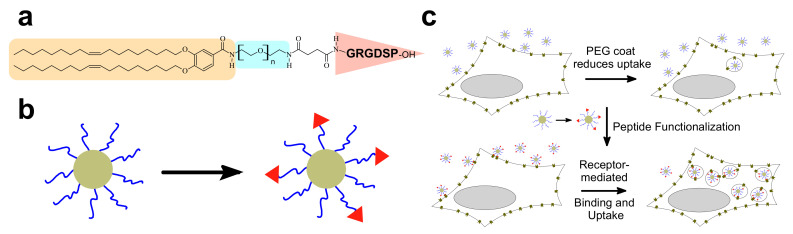
(**a**) Structure of the RGD-PEG2K-lipid as an example of a ligand-PEG-lipid. The peptide ligand is highlighted with a red triangle, the lipophilic tails in beige, and PEG in blue. (**b**) Schematic depiction of ligand-tagging of CL–NA NPs. (**c**) PEGylation reduces cellular uptake of NPs, reducing efficacy. Functionalization of the distal end of a PEG-lipid with an appropriate ligand induces receptor-mediated binding and increases cellular uptake (and thus efficacy) in cells expressing the peptide’s receptor [180].

Below, we describe two complementary strategies that can be used for partial recovery of the TE of PEGylated CL–DNA NPs (Figure 13): (1) low pH-induced dePEGylation to promote endolysosomal escape (Figure 14) [179,188,189] and (2) conjugating affinity targeting ligands to the PEG to favor uptake in target cells (Figure 15) [180].

### 8.1. Low pH-Induced dePEGylation

The tail length affects the rate of desorption of PEG-lipids from CL–NA NPs, and careful choice of tails can tune NP stability in vivo [190]. However, other strategies are required at high PEG densities. One such strategy involves incorporating acid-labile bonds between the tails and the PEG moiety of the PEG-lipids, to induce dePEGylation at the low pH (≈5) of late endosomes. The acidification that is part of the maturation process of the endosome cleaves the PEG chains from the lipid tails and thus removes the PEG-coat of the NP. This enhances TE because it “switches on” the electrostatic attractions required for endosomal escape via membrane charge density-promoted activated fusion [17] by “unmasking” the charge of the NP (Figure 14b). The chemistry of the acid-labile bond is a crucial aspect of this strategy because it allows fine-tuning the pH ranges where cleavage occurs [191]. A successful example is a PEG-lipid (termed HPEG2K-lipid; PEG MW 2000 Da) bearing an acid-labile acylhydrazone bond between the lipid tails and the PEG chain (Figure 14a) [179]. The HPEG2K-lipid is stable at pH = 7, but the PEG chain is cleaved from the lipid tails at pH = 5. Figure 13 shows that complexes containing 10% HPEG2K-lipid (green diamonds) exhibit partial recovery of TE when compared to complexes containing 10% PEG2K-lipid (blue triangles) [179].

### 8.2. Affinity Targeting of PEGylated CL–DNA Complexes

PEGylation strongly reduces nonspecific attractive interactions of CLs and CL–NA NPs with the cell surface. This reduces efficacy but also opens the opportunity of specific targeting with affinity ligands such as peptides, antibodies, and aptamers. For example, uptake of PEGylated CL–DNA complexes in cultured cells can be increased by tagging the NPs with RGD peptides [192,193,194,195] that bind to the cell surface integrins expressed on the target cells (Figure 15). Figure 15a shows the GRGDSP-PEG2K-lipid [180,196], a PEG-lipid with a distally attached linear RGD peptide that shows high affinity for α_v_β_3_ and α_v_β_5_ integrins. Incorporating this peptide-PEG-lipid into NPs instead of the DOB-mPEG2000 lipid (Figure 3) results in NPs that are decorated with the peptide ligand (Figure 15b). While highly PEGylated NPs are sparsely taken up by cells (Figure 15c, top), the ligand-tagged NPs enter integrin-positive cells in greater number by receptor-mediated endocytosis. Live-cell imaging with quantitative colocalization analysis directly confirmed this increased NP uptake [180]. The increased internalization was paralleled by improved cellular delivery of the DNA payload: transfection experiments showed an increase in TE (Figure 13b).

### 8.3. Organ- and Disease-Specific Targeting Peptides

In addition to increasing efficacy by promoting uptake in cultured cells, peptide ligands may be used to target specific healthy and diseased tissues in vivo [197,198]. The systemically accessible molecular landscape on endothelial cells bears tissue type- and status-dependent signatures. This vascular heterogeneity can be explored in an unbiased manner by in vivo screening of phage libraries that display random, genetically encoded peptide sequences [199]. This screening process consists of injecting the phage library into the systemic circulation, rescuing the phage from the target tissue, and repeating the process to derive a phage pool with the ability to home to the intended target tissue [200]. The in vivo phage display approach has yielded a variety of homing peptides specific for normal organs as well as for tumor vasculature and tumor cells [198,201,202,203,204,205,206,207,208,209,210]. Recent advances in the approach, most notably the application of high-throughput sequencing and development of bioinformatics tools to analyze the resulting data, allow for streamlined, robust, and reproducible identification of homing peptides [211,212] and mapping of the vascular diversity.

In vivo phage display and chemical optimization of the discovered peptides for increased stability and affinity have greatly expanded the spectrum of peptide ligands and potentiated their binding since the initial discovery of the RGD-motif [192,193,194,213]. An example of chemical optimization is head-to-tail cyclic RGD peptides (e.g., c(RGDfK) [214,215]) with high affinity (up to 1000× larger than linear RGD [216,217,218]) for the α_v_β_3_ integrin overexpressed in many cancer cell lines in vitro and in malignant lesions in vivo. Another example is the development of stabilized versions of the fibrin-targeting CREKA peptide containing nonproteinogenic amino acids [219].

Importantly, and of translational relevance, the homing peptides are typically not species-specific—most of the homing peptides bind the corresponding human epitopes as well. Whereas homing peptides typically use docking interactions for accumulation in endothelial cells of target tissues, some homing peptides are tissue penetrating: they specifically recognize the endothelium of target vessels, extravasate, and penetrate deep into the extravascular tissue [220,221,222,223,224]. This class of homing peptides is particularly useful for targeting cells in the tissue stroma. One such tumor penetrating peptide, iRGD (sequence: disulfide-cyclized CRGDKGPDC) [221,222,223,225], is undergoing clinical testing for precision delivery of paclitaxel albumin nanoparticles (Abraxane) and gemcitabine to pancreatic cancer lesions (ClinicalTrials.gov Identifier: NCT03517176, “CEND-1 in combination with Nab-paclitaxel and Gemcitabine in Metastatic Pancreatic Cancer”).

Another example of a tumor penetrating peptide is TT1 (sequence: disulfide-cyclized CKRGARSTC) and its linear version linTT1 (AKRGARSTA). These peptides target p32 (HABP1/gC1qR), a protein that is upregulated and aberrantly expressed on the surface of malignant cells and activated macrophages in solid tumors and in atherosclerotic plaques [226,227,228,229,230,231,232,233].

In light of the current interest in CL–NA NPs for vaccine development, it is worth mentioning another recently identified targeting peptide, mUNO (sequence: CSPGAK). This peptide targets CD206 (known as the mannose receptor) on mouse and human cells when coated on polymeric nanoparticles [234,235,236]. The mannose receptor is widely used for delivering material to antigen-presenting dendritic cells for vaccine applications [237,238,239].

Receptors, i.e., the binding partners of homing peptides, can be identified using different biochemical approaches, such as affinity chromatography and proximity biotin ligation-based assays. Expression and accessibility of peptide receptors vary not only between different tissues and organs but also between healthy and diseased tissues [201,202,203,204] and between particular cell populations within the target tissue [234]. Examples include certain growth factor receptors (e.g., VEGFR2, neuropilin-1), cell adhesion molecules, and intracellular proteins (e.g., p32, nucleolin) aberrantly expressed at the cell surface in tumor vessels and malignant cells, CD206 expressed on M2 macrophages [234] and certain tumor-associated extracellular matrix isoforms [240,241].

### 8.4. Peptide Ligands Promote Tumor Targeting and Penetration of CL–DNA NPs In Vivo

**Figure 16:**
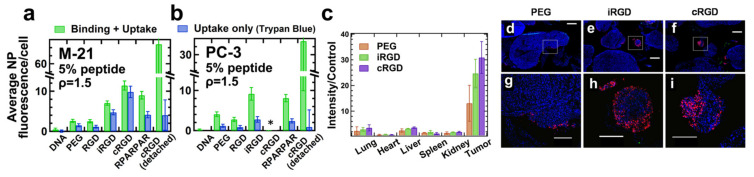
Peptide-tagging for specific targeting of CL–DNA NPs in vitro and in vivo. NPs were formulated at a lipid/DNA charge ratio of 1.5 and a molar ratio of 10/70/10/5/5 of MVL5/DOPC/cholesterol/PEG-lipid/x, where x = PEG-lipid (control) or peptide-PEG-lipid. (**a**,**b**) Fluorescence from bound and internalized NPs containing labeled DNA in two cell lines (M21 and PC-3) measured by flow cytometry. The graphs compare several tagged (peptide-PEG-lipid) and untagged (PEG-lipid only) NPs with free DNA (no lipid) as a control. Binding and uptake were differentiated by the addition of Trypan Blue, a membrane-impermeable dye that quenches the fluorescence of NPs outside the cells. NPs tagged with cRGD detached a large number of cells from the substrate. These cells were measured separately. *: too few cells remained attached to allow measurement. (**c**) In vivo biodistribution of intraperitoneally (*i.p.*) administered CL–DNA NPs. Mice bearing intraperitoneal MKN-45P tumors were *i.p.* injected with either PBS (control) or ~0.5 mg of CL–DNA NPs. After 24 h the tumors and organs of interest were excised and the fluorescent signal from the Cy5-labeled DNA was imaged (inset) and quantified (bars; normalized to control; *n* = 3). The vast majority of the fluorescent DNA is found in the tumor, and peptide-tagged NPs show higher selectivity for the tumor than untagged NPs. (**d**–**i**) Confocal microscopy images showing CL–DNA NPs in sections of the tumor nodules. Parts (**g**–**i**) are enlarged views of the marked areas in parts (**d**–**f**), respectively. Cy5 (DNA-label, i.e., NPs): red, DAPI (cell nuclei): blue. Tumor nodules from mice treated with untagged (control) PEG2000-lipid NPs (**d**,**g**) show NPs on the nodule surface, while iRGD- (**e**,**h**) and cRGD-tagged NPs (**f**,**i**) penetrated into the tissue of smaller tumor nodules (diameter ~300 μm). Scale bars: 500 μm (**d**–**f**) and 200 μm (**g**–**i**). Adapted from [242], Copyright 2018, with permission from Elsevier.

Recent work used flow cytometry to screen peptides and compositional parameters of CL–DNA NPs (such as the lipid/DNA charge ratio and peptide coverage) for optimal in vivo performance (Figure 16a,b) [242]. The study investigated vectors tagged with linear GRGDSP (“RGD”), the cyclic iRGD tumor penetrating peptide (above), cRGD (c(RGDfK), above)), and RPARPAR. RPARPAR is a prototypical example of peptides containing an active C-end Rule (CendR) motif, R/KXXR/K. This motif, which has to be exposed at the C-terminus for activity, is shared between all tumor penetrating peptides [221]. Interestingly, intermediate peptide coverage (e.g., 5 mol% peptide-PEG-lipid + 5 mol% PEG-lipid rather than 10 mol% peptide-PEG-lipid) was optimal for in vitro binding and internalization in receptor-positive cells [242]. At a low lipid/DNA charge ratio, binding and internalization were moderately high for NPs tagged with iRGD and RPARPAR and highest for those tagged with cRGD (Figure 16a,b) in multiple cell lines in vitro. However, the higher positive charge of RPARPAR resulted in higher nonspecific attachment, showing that ligand charge is an additional important parameter to consider when designing targeted NPs.

In vivo biodistribution experiments, in which NPs tagged with iRGD and cRGD peptides were systemically administered to mice bearing peritoneally disseminated human gastric cancer (with a PEG-lipid control) revealed that both peptide-tagged NPs homed to the tumors at higher levels than the control NPs (Figure 16c). Importantly, in tumors, the peptide-tagged NPs also extravasated and penetrated into malignant tissue, in particular into small nodules (which are harder to remove surgically and thus a desirable target for therapeutics) (Figure 16e,f,h,i) [242].

## 9. Cationic Liposomes for The Delivery of Hydrophobic Drugs

Aside from NA delivery, cationic liposomes have received intense interest as carriers for other classes of therapeutics, in particular hydrophobic cancer drugs (Figure 1) [243,244,245,246,247,248,249]. Many of the principles underlying structure–activity relationships of CL–NA assemblies (such as the effect of lipid shape on assembly structure) that have been outlined in the previous sections are also relevant in this context, even as some intriguing differences exist (e.g., with respect to the effect of PEGylation, see below).

A prominent example of a hydrophobic cancer drug is paclitaxel (PTX), which is among the most widely used chemotherapy drugs to treat ovarian, breast, lung, pancreatic, and other cancers [250,251,252,253,254,255,256,257,258]. Because of their hydrophobicity, these drugs reside within the lipid bilayer rather than in the aqueous interior of the liposome (Figure 1, red spheres). This means that they will remain associated with the lipid membranes upon formation of CL–NA complexes, resulting in the exciting potential to combine them with NAs into therapeutics with dual action [259].

Because of their low water solubility, hydrophobic drugs such as PTX are not effective unless formulated with a carrier. Currently used formulations of PTX (such as Taxol^®^ and Abraxane^®^); frequently cause hypersensitivity reactions and/or deliver PTX non-discriminately throughout the body [260,261,262]. Therefore, the development of liposomal formulations of PTX with high efficacy is an extremely active field of research [263,264,265,266,267], including in clinical trials [249,267]. Cationic liposomes have been shown to target tumor neovasculature [244,245,246,268,269] and are readily internalized by cells. This makes cationic liposomes desirable vectors for hydrophobic drugs because molecules such as PTX have to reach the inside of cells to unfold their activity.

**Figure 17:**
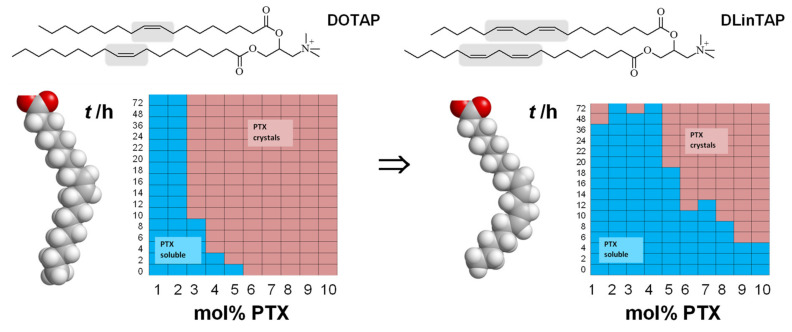
**Top:** Structure of DOTAP, with oleoyl (C_18_ with a single cis double bond) tails, and the corresponding lipid DLinTAPwith linoleoyl tails (with an additional cis double bond). **Bottom:** Space-filling molecular models of the ground-state structure of the lipid tails and PTX solubility kinetic phase diagrams for the corresponding DOTAP/DOPC and DLinTAP/DLinPC formulations. Formulations of increasing PTX content (*x*-axis) were monitored over time (*y*-axis) for PTX crystallization (red color). Blue color indicates the absence of PTX crystals. See Figure 3 for the structures of DOPC and DLinPC. Solubility phase diagram data reprinted with permission from [109].

A common feature of liposomal formulations of PTX is their relatively low loading capacity, at 3 mol% of the lipid content. Improving the PTX loading capacity of liposomal carriers requires enhancing PTX solubilization within the membrane [270]. Recent work showed that there is great potential for achieving this by modifying the lipid tails [109]. PTX membrane solubility in cationic liposomes with tails containing two *cis* double bonds (linoleoyl tails) was significantly increased compared to tails with one (oleoyl tails) double bond: 8 mol% PTX in the former remained soluble for approximately as long as 3 mol% PTX (the above-mentioned membrane solubility limit) in the latter (Figure 17). Comparison with DOPE-containing formulations suggests that the increase in solubility is likely not caused by the structural change from bilayers to inverse cylindrical micelles (Figure 4) but rather by the enhanced molecular affinity between lipid tails and PTX. Importantly, the efficacy of the PTX-loaded CLs was unaffected by changing the lipid tails in one cell line; in another cell line, the efficacy was even increased two-fold. These findings demonstrate the potential of chemical modifications of the lipid tails: liposomal PTX carriers with increased PTX solubility, at maintained or even increased efficacy, reduce side effects and costs because they require less lipid to deliver a given amount of PTX.

**Figure 18:**
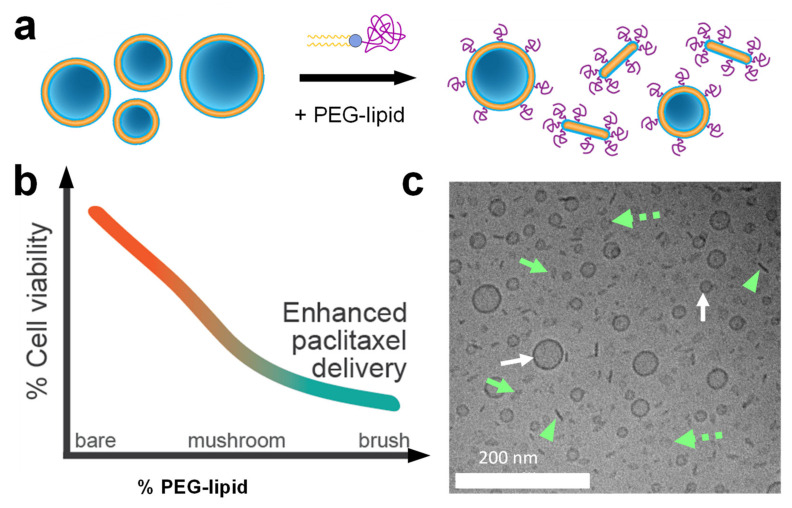
(**a**) Schematic illustration of the structural transitions observed upon PEGylation of PTX-carrying CLs. Unilamellar vesicles of varying sizes are replaced by small vesicles and discoidal micelles (bicelles). (**b**) Cytotoxicity of PTX-carrying CLs as a function of increasing PEGylation (at a constant amount of PTX). The efficacy of the CLs against cancer cells increased (cell viability decreased) with the extent of PEGylation. (**c**) Cryogenic electron microscopy image of a formulation of DOTAP/DOPC/PEG2000-lipid/PTX at a molar ratio of 50/37/10/3. Small vesicles of varying size (white arrows) and edge-on (green arrowhead), tilted (green arrow), and top-down (green dashed arrow) views of discoidal micelles are discernible. Adapted with permission from [112]. Copyright 2020 American Chemical Society.

As mentioned earlier, PEGylation of CL-based NA carriers is necessary for in vivo applications but diminishes efficacy. Surprisingly, recent work showed that PEGylation of CL-based carriers of PTX enhances, rather than diminishes, delivery efficacy and cytotoxicity against human cancer cells (Figure 18a,b). This unexpected enhancement occurs even at low PEG-lipid content, when the PEG chains are in the transition regime between the mushroom and brush conformations. Cryogenic TEM showed that PEGylation leads to a mixture of nanometer-scale vesicles and anisotropic micelles (Figure 18a,c). The formation of disk-shaped micelles at sub-monolayer concentrations of the PEG-coat was unexpected; it had previously been assumed that vesicles would incorporate such relatively small amounts of PEG-lipid without modifying their shape. Confocal microscopy and flow cytometry revealed significantly enhanced cellular uptake of PTX for PEGylated (vesicles and disks) compared to bare PTX-loaded liposomes. This suggests that steric stabilization can facilitate NP entry into cells via distinct size-dependent endocytic pathways that are inaccessible to larger liposomes and aggregates. This study highlighted the value of understanding how PEGylation alters NP shape and structure, and thus NP efficacy, to design next-generation stealth drug carriers. Importantly, these PEGylated liposomes will allow integrating active cell-targeting strategies for delivery in vivo.

## 10. Concluding Remarks

As the range of applications of cationic liposomes in the delivery of therapeutics further matures and grows, it becomes ever more important to develop a fundamental science base that can inform the rational design of these self-assemblies. We have shown in this review that it is essential to consider principles from soft matter and biophysics and lipid and colloid science when trying to understand the structure–activities of the cationic and neutral lipids, because the lipids exert their function as an assembly of molecules. The results of ongoing studies investigating the barriers to targeted delivery and the mechanisms of intracellular uptake, transport, and release of these vectors and their interactions with cell components will continue to inform the design and synthesis of optimal lipid carriers of NAs.

Even as the medical applications are just beginning, cationic liposome-based vectors of nucleic acids for gene delivery, gene silencing, and gene editing have found myriad applications in fundamental and applied biological research. Further advances in the delivery systems will only add to these applications, be it in studies of chromosome structure and function through the development of efficient vectors for very large DNA constructs, or in molecular biology studies through improvements in the ability to efficiently deliver NAs into hard-to-transfect cell lines such as T-cells, macrophages and dendritic cells. Other important frontiers of the field are selectively targeting tissues beyond the liver (patisiran) and local delivery (mRNA vaccines). Finally, for cancer therapeutics, the ability of CLs to simultaneously deliver both NAs and hydrophobic drugs in combination therapies holds great promise.

## Data Availability

Not applicable.
